# A socio-cultural study of traditional healers role in African health care

**DOI:** 10.1186/s13027-019-0232-y

**Published:** 2019-06-20

**Authors:** C. C. Asuzu, E. O. Akin-Odanye, M. C. Asuzu, J. Holland

**Affiliations:** 1 Department of Counselling and Human Development Studies, University of Ibadan, Ibadan, Nigeria; 2 Department of Radiotherapy, University College Hospital, Ibadan, Nigeria; 3 Department of Family Medicine, University College Hospital, Ibadan, Nigeria; 4 Department of Community Medicine, University College Hospital, Ibadan, Nigeria; 5 Department of Psychiatry & Behavioral Sciences, Memorial Sloan Kettering Cancer Centre, New York, USA

**Keywords:** Cancer, Patients, Traditional healers, Collaboration, Spiritualists

## Abstract

**Background:**

There is a widely held view that a major cause of delay in diagnosis of cancer at an early stage in Africa is the fact that many patients consult traditional healers first and are often treated by them until curative treatment cannot be undertaken.

**Purpose:**

This qualitative study aimed at identifying cancer patients who have used traditional healers and their traditional healers’ understanding of cancer, its causes and treatment, patients’ motivations for patronizing traditional healers and their opinion about collaboration between physicians of western medicine and traditional healers as part of overall health care system.

**Methods:**

Ethical approval was obtained from the UI/UCH IRB. Twenty people participated in the study, nine cancer patients, six traditional healers and five faith based healers using three focused group discussions employing a semi structured interview.

**Data analysis:**

The audio taped recorded responses to the semi structured questions were transcribed and thematically analyzed. The themes that emerged from focus group discussions with the patients and healers relate to the meaning of cancer, the causes of cancer (due to satanic attack), the cure for cancer (being possible through prayers to God and use of herbs), reason for using traditional healers (financial consideration, convenience, incorporation of their spiritual beliefs and recommendation by others) and need for collaboration between orthodox medicine and traditional healers.

**Conclusion:**

Patients and alternative healers have a poor understanding of the causes and treatment of cancer. They desire to seek a closer collaboration between healers and western physicians to enhance better care for cancer patients. This has implication for policy makers who will facilitate the relationship in order to control cancer care and improve the quality of care of cancer patients in Nigeria.

## Background

Nigeria comprises an assortment of dissimilar cultural entities made up of more than 250 ethnic groups. The three key ethnic groups are Yoruba, Hausa/Fulanis and Igbo located in the southwest, north and southeast of Nigeria respectively. Each of the different ethnicities has its own indigenous ways of ensuring the health of its people. Hence, an enduring component of the primary healthcare system in Nigeria is the alternative healthcare system, which is made up of the traditional and faith based healers. Despite the presence of western medicine in the primary healthcare system in Nigeria, it has been observed that traditional medicine in whatever form is being used and it is still popular among 70% of the population [6].

A prevalence rate of herbal use of 41% with 31%, combination of orthodox and traditional medicine was reported in a nationwide survey in Nigeria [13]. Alternative medical treatment was found to be popular among 35.7% of respondents in a study in Southwest Nigeria even when they were aware that it may be injurious to their health [5]. This may not be unassociated with the recurrent adverts of herbal remedies on most newspaper in Nigeria [11]. Also, it is opined that alternative medicine is natural, cheaper, more accessible, acceptable and effective [5, 7, 10, 12].

It is often opined that the use of alternative healers has led to cancer patients delay in presentation at the hospital for treatment as patients tend to visit alternative healers first for treatment [15, 17]. Among cancer patients however, Ezeome [9] reported that 82.3% of cancer patients in Enugu reported for initial evaluation at a modern health facility while 17.5% reported first to alternative practitioners. Other factors apart from first presenting to alternative practitioners, such as ignorance, being young, illiterate, poverty, residing in a rural area, superstition, denial, fear of mastectomy and unavailability of treatment facilities [3, 18] could also account for late presentations at hospital. A finding worthy of note in eastern Nigeria, is that more than 50% of fairly high literate patients waited more than six months after noticing a breast mass before going to the hospital [4].

Not much has been done in Nigeria with regards to understanding traditional healers’ conception about cancer. To create a foundation for the working out of modalities for the collaboration between western and traditional medicine healers in Nigeria, this study addressed at the qualitative level what the patients understand and believe about traditional healers and what they understand and would find helpful in their practice.

## Purpose of the study

This study was designed to assess cancer patients and their alternative (traditional and faith based) healers’ conception of cancer, its cause(s), treatment effectiveness, as well as their stance on collaboration between alternative healers and orthodox medicine physicians.

## Method

### Study area

Data for this study was collected from Ibadan Oyo State and Ipetu-Remo in Ogun State both in Southwest Nigeria.

### Procedure

Ethical approval was obtained for this study from the UI/UCH IRB. Qualitative method was used in gathering data through three focused group discussions. The three focused group discussions were with 9 cancer patients, 6 traditional healers and 5 faith based healers. The focused group discussion with the cancer patients took place at the Radiation Oncology Department of University College Hospital, Ibadan while that with the faith based healers took place at a church in Ibadan and the discussion with the traditional healer took place at the residence of one of the traditional healers in Iperu-Remo in Ogun State. Two different semi-structured interview schedules were used for the cancer patients and the alternative healers. The sessions were audio tapped after informed consent was obtained from the participants.

### Data analysis

Text data collected from the transcribed audio recording obtained during the interview sessions were thematically analyzed. Themes which emerged based on the recurrence of responses to semi-structured questions amongst the study participants were reported for the groups.

## Results

### Demographics

The 9 cancer patients that participated in the focused group discussion had earlier disclosed that they had patronized alternative healers because of their ailment either before or after receiving treatment at the University College Hospital, Ibadan. Some of the cancer patients gave the addresses of the alternative healers they patronized. Two alternative healers (1 traditional and 1 faith based) were approached and asked to invite their colleagues to participate in the study.

A total of 20 participants made up of 9 cancer patients, 6 traditional healers and 5 faith based healers participated in this study. The cancer patients who were made up of 8 females and 1 male were all married with an age range of 39 years to 66 years and a mean of 51 years. Most of patients had secondary school education. With regards to their cancer types, 5 had cervical cancer, 3 had breast cancer and 1 had throat cancer.

The 11 alternative healers (6 traditional healers and 5 faith based healers) were all married male with an age range of 34 to 84 and a mean of 55.09. The traditional healers’ educational qualification ranges from no formal education (1), primary education (2), secondary education (2), tertiary education (5) and Grade 3 education (1). Their years of practice as healers ranged between 8 to 61 years.

### Themes generated from the focused group discussions

The themes generated from the focused group discussions are categorized in terms of the patients and alternative healers’ perception in relation to:

1. Their conception of cancer and its causes
2. Treatment modalities and comparative treatment effectiveness between alternative and orthodox medicine
3. Their stance on collaboration between alternative healers and orthodox medicine physicians

### Alternative healers and cancer patients’ conception of cancer and its causes

The traditional healers could not describe what cancer is but they alluded to the causes of cancer. One of the healers said “cancer is caused by eating foods like cassava, poor personal hygiene and infection with bacteria” and another said “cancer is caused by viruses and body impurities”. The traditional healers thus attributed the cause of cancer to more physical and pragmatic causes. A faith based healer in describing what cancer is said “cancer is a life, it is a spirit of infirmity”. While one of the faith based healers did not know the cause of cancer said “I wouldn’t know because I am not a medical practitioner”; others said cancer could be caused by “ignorance and satanic attack”, “diet and it could be an attack”; “demonic attack, overwork and stress”. A recurring theme with regards to the cause of cancer is ‘attack’ denoting more of a spiritual cause. Amongst the cancer patients’ group, the only male cancer patient described cancer “… as a multiplication of cells that refuse to die”. Other representative responses include “I just know that it is a deadly disease”. Others did not know what cancer is and made comments like “I don’t know”, Patients’ responses to what causes cancer vary. “Cancer is a parasite which can enter the body through the air we breathe in, what we eat or through sex and family planning”. “It is only God that knows what causes the cancer”, “In my mind I also think environment and lifestyle can cause cancer”. The cancer patients’ attribution of the cause of cancer had a mix of spiritual and environmental/lifestyle factors.

### Treatment modalities and comparative treatment effectiveness between alternative and orthodox medicine

Treatment modalities used by the traditional healers include herbs for drinking, preparations for skin application, divination, incantation and sacrifice. The faith based healers use the spoken word, prayer, holy water, anointing oil and fasting on behalf of the patient. Cost for treatment per visit ranged from N2,000 to N5,000 for the traditional healers while that for all the faith based healers was free. A representative comment in this regard among the faith based healers is “the power to heal was freely given by God, so healing is free but if patients recover and decide to give anything in appreciation, it is accepted”. With regards to healers’ perception on effectiveness of alternative medicine compared to orthodox medicine, the traditional healers did not have a uniform opinion as 2 said it was more effective, 2 said about the same, 2 said less effective. Majority (4 out of 5) of the faith based healers however said their method was more effective while 1 was undecided Most of the patients indicated that they had used one form of alternative treatment or the other but believe more in prayer. Here is a patient’s account “I started with going to different churches but none of them told me I had cancer except a man that told me I had a serious sickness in my stomach and I should go to the hospital. It was when I went to the hospital I was told I had cancer of the womb”. As to the efficacy of alternative treatment, a patient said “You can receive healing from any pastor that is a true man of God”, “I believe that traditional healers can also cure cancer with herbs, especially with the use of things like garlic, ginger, etc”. A patient said “I’m still insisting that in terms of cancer I doubt if there is anything a native doctor can offer”. The patients seem to hold the opinion that orthodox medicine is more effective than alternative medicine if it is used with prayer.

### Alternative healers’ and cancer patients’ stance on collaboration between alternative healers and orthodox medicine physicians

All the alternative healers would like to collaborate with orthodox doctors in treating cancer patients. However while all the traditional healers would be willing to send cancer patients for hospital diagnosis and staging only 2 of the faith based healer would be willing to do so. The theme in the discussion with the faith based healer is this regard is “If I refer them to the hospital first, then they may lose faith in me and my God”.

From the patients’ perspective, “There is need for collaboration for progress as a tree cannot make a forest and two heads are better than one. The traditional healers use herbs, more researches can be carried out with the herbs used by the traditional healers to find out the active components”, Another patient noted that “In those days before the white man’s medicine came, there must have been cancer and the traditional healers most have been treating them because our forefathers lived longer than we are now living unless if we say there was no cancer in those day, hence the native doctors must have something to offer medicine in the treatment of cancer.” Another patient thinks that the native healers contribute significantly to curing cancer and explained that, “when I developed a wound on my breast, the native doctor used both herbs and ampicillin on the wound and I felt better”.

Patients generally believe their spirituality should be incorporated into their cancer care. “I believe chaplaincy is good to strengthen the faith of the patients so that they encourage the patient to hang in there and not give-up but I do not subscribe to the use of concoctions while receiving treatment”. Another patient doesn’t think that the hospitals should necessarily involve the chaplains, but believe the doctors should be involved personally. “When doctors engage in prayers before treating their patients, treatment outcomes will be enhanced. All health care professionals should dedicate time to build up spiritually before starting work of caring for their patients”.

## Discussion

Most of the patients and traditional healers in the current study did not really know what cancer is. Only one patient could relate cancer with cells growth and multiplication while none of the traditional healers knew what cancer was. As to the cause of cancer while most of the patients believe it is most likely caused by a spiritual attack, the traditional healers mostly believed that cancer could be due to infection with bacteria and viruses, stress, eating of certain foods like cassava and poor personal hygiene. Similar misconceptions about the meaning and cause of cancer have been reported in other related studies [1, 2]. The faith based healers describe cancer as an infirmity and as mainly caused by spiritual attack. Most patients in this study have used either herbal or spiritual remedies for their cancer before going to the hospital. Some of them only go to the hospital because the traditional treatment was not as effective as they had hoped or because the healer advised it.

There was a mixed opinion as to the efficacy of alternative treatment for cancer amongst the patients in this study. While majority of the patients believe traditional healers who use herbs can cure cancer a few doubt their ability to do so. Others use a mix of faith based healing therapy with the orthodox treatment. All the patients unanimously believe in the efficacy of prayers in curing cancer. Hence some of the patients suggest that chaplaincy should be integrated into the overall cancer care. Indeed, it is almost impossible to divorce our patients’ healthcare from their spirituality. Cancer patients have been reported to use a variety of religious and spiritual resources, such as personal faith and prayer, relationship with God, prayers from fellow church members, pastor/priest or leader of faith, reading the bible, attending religious services, meditation, spiritual retreats as means of coping with their health conditions [16].

In the light of the burden of a cancer diagnosis and the danger in delaying the commencement of effective treatment, there is need to focus on how the dual traditional and orthodox medical systems can work together in collaboration to optimize patient healthcare. The alternative healers and cancer patients in this study desire collaboration with the practitioners of the orthodox healthcare system. Most healers want to learn how to treat illness more effectively, so they are highly motivated to cooperate with the modern health sector [8]. Razali [14] has proposed a three-step process for achieving this collaboration. Firstly, he proposed that the government should formally recognize the traditional healers as having an important role to play in the health system. Secondly, traditional healers should be better organized in terms of forming a body to register and monitor the activities of their members. Finally, a sort of retreat could be organized in which traditional and orthodox health workers have opportunities to dialogue on their respective roles, expertise and limitations. This will enhance communication between the two practitioners.

## Conclusion

This study has advanced our knowledge about traditional healers and has revealed that they would like to learn more about cancer and to collaborate with western medicine physicians. This study is an effort to understand patients’ perceptions and alternative healers’ knowledge regarding cancer and its causes. A clear knowledge gap exists in alternative healers’ conception of cancer. If they will ever be able to make appropriate referrals, then there will be a need to close this gap through health education that can be gained from a mutual relationship with orthodox medicine practitioners. Legislation is needed by policy makers to facilitate the relationship in order to control cancer care and improve the quality of care of cancer patients in Nigeria.

## Data Availability

The datasets generated and analyzed during the current study are available with the Principal investigator.
